# A data-driven approach shows that individuals' characteristics are more important than their networks in predicting fertility preferences

**DOI:** 10.1098/rsos.230988

**Published:** 2023-12-20

**Authors:** Gert Stulp, Lars Top, Xiao Xu, Elizaveta Sivak

**Affiliations:** ^1^ Department of Sociology, University of Groningen, Grote Rozenstraat 31, 9712 TS Groningen, The Netherlands; ^2^ Inter-University Center for Social Science Theory and Methodology, University of Groningen, Grote Rozenstraat 31, 9712 TS Groningen, The Netherlands; ^3^ Netherlands Interdisciplinary Demographic Institute (NIDI-KNAW), Lange Houtstraat 19, 2511 CV Den Haag, The Hague, The Netherlands

**Keywords:** fertility, personal network, social influence, predictive ability, LASSO regression

## Abstract

People's networks are considered key in explaining fertility outcomes—whether people want and have children. Existing research on social influences on fertility is limited because data often come from small networks or highly selective samples, only few network variables are considered, and the strength of network effects is not properly assessed. We use data from a representative sample of Dutch women reporting on over 18 000 relationships. A data-driven approach including many network characteristics accounted for 0 to 40% of the out-of-sample variation in different outcomes related to fertility preferences. Individual characteristics were more important for all outcomes than network variables. Network composition was also important, particularly those people in the network desiring children or those choosing to be childfree. Structural network characteristics, which feature prominently in social influence theories and are based on the relations between people in the networks, hardly mattered. We discuss to what extent our results provide support for different mechanisms of social influence, and the advantages and disadvantages of our data-driven approach in comparison to traditional approaches.

## 1. Introduction

The rapid decline in fertility in the last few centuries is at least partly due to social influences on reproductive behaviour [1–3]. People's desired and actual number of children are shaped by the preferences and actions of others. This is supported by diverse sources of evidence. Early research has focused on the spread of fertility decline across regions [4,5], revealing that culturally similar regions tend to have similar fertility rates, regardless of economic factors. A different line of evidence comes from studies investigating the impact of certain individuals or groups within one's network. Research has shown that kin [6–9], high-school class mates [10], friends [11] and colleagues [12,13] influence when people have children and how many.

Qualitative research has been successful in identifying mechanisms of social influence [14,15]. One such mechanism is *social learning*; people learn from others, for example, about the right time to have children and how to combine work and family [16]. *Social contagion* is the process in which preferences are shaped through emotional states rather than explicit learning [17]. For example, increased broodiness after having been around (cute) children [18,19]. A third mechanism is *social support*: family and friends can provide emotional, instrumental or financial support that facilitates fertility behaviour [11,16,20–22]. These people may equally well exert some pressure to have children or to not have children [11,17,23–26], which is referred to as social *pressure*.

There is thus a substantial body of evidence for social influences on fertility [17]. Yet, prior research faces limitations due to challenges in gathering network data. This research often examines the impact of specific groups within networks (e.g. only colleagues). Furthermore, the number of people in the network for whom data is collected is rather small, often focusing on close relationships. Moreover, extensive network data mostly exist for highly selective samples. Below, we highlight the advantages of collecting data from larger personal networks to address these limitations.

### 1.1. The value of personal networks

Personal (or egocentric) network data collection is a promising way of establishing social network effects on fertility. In such data collection, people are first asked to list a number of people in their network, and then to provide information about those people. In the personal network literature, the person who reports about the network is often referred to as *ego*, the people in the personal network as *alters*, and the relationships between ego and the alters and the relationships between the alters as *ties*. For example, people can be asked about their friends (*alters*), the quality of their relationship with these friends (*tie strength*), and their behaviour (*alter characteristics*). This allows addressing different social influence processes. First, the importance of the strength of the relationships can be assessed. The closeness of one's relationship with parents and kin will likely shape the support one receives or the normative pressure one perceives [17,23,24,27]. Strong ties with people who recently had children may also nudge towards pro-natal sentiments [11]. Second, the importance of the *composition* of the network can be assessed. Composition refers to the (diversity in) characteristics of the people in the networks, and is often reflective of the content and resources that are available in the network. For example, more kin in a network can mean more support when raising children [8,9,28,29].

Personal networks can also provide insights into the importance of network *structure*, when information is available on the relationships between people in the network (*alter–alter ties*). For example, the density of a network is defined as the number of ties between people relative to the total number of possible ties. Density is believed to have important effects on social influence processes, because it shapes how information flows through networks. In dense networks, alters are likely to share similar views and are better able to collectively influence ego and exert pressure to conform [30–32]. In sparse networks, alters likely have different ideas, and novel ideas flow more freely through the network [33,34]. Collecting information on ties between alters is rare, which is why only a handful of studies have been able to assess network structural effects on fertility outcomes (see [11,30,31] for notable exceptions).

An important consideration in personal network data collection is the number of alters to ask about. Asking for many alters in a survey and assessing characteristics of these alters and the alter–alter ties presents a significant burden on respondents in both time and repetitiveness of answering questions [35–37]. This is why personal networks are often limited in size. However, small(er) personal networks are problematic when the composition and structure of the network are of interest, because these can only be reliably assessed when the networks are of sufficient size. Previous research suggests that networks of around 20–25 people are sufficient [35,36].

### 1.2. A data-driven approach to network effects

Personal network data thus allow calculating many different compositional and structural characteristics, which relate to different mechanisms of social influence [11,15]. Existing research on social influences on fertility has largely followed the traditional theoretical and statistical approach dominant in the social sciences to focus on a small number of variables (e.g. the percentage of kin in the network and perceived social support), provide a theoretical model of how these variables are (causally) related, and use inferential statistics to assess support for this model. This approach has led to a rich body of empirical work. Here we take a different, data-driven approach. Before explaining why a data-driven approach is particularly useful in assessing network effects on fertility, we first explain what a data-driven approach entails and its advantages.

Recent research convincingly argues that data-driven methods, which are often focused on prediction, can advance research in the social sciences [38–41]. Prediction is sometimes seen as in opposition to understanding, but it is better conceived as a complementary form of understanding to the traditional theory-driven approach [39,42,43]. There are certainly advantages to a focus on prediction. First, out-of-sample prediction is an easy-to-interpret and comparable effect size that allows for a comparison between models and even between statistical techniques [43,44]. Second, because it measures how well a theory (or model) does on unseen data, it is a measure of how well our theories do in practice [44,45]. Third, it is less susceptible to researcher degrees of freedom and questionable research practices as, for instance, the *p*-value [43].

Data-driven approaches (e.g. machine learning methods, cross-validation [46]) are typically designed to optimize out-of-sample prediction [47]. The strength of these approaches relative to traditional statistical practices is that they prevent both underfitting (omitting important variables in the model) and overfitting (including too many variables in the model, some of which will be associated with randomness in the data), and that they can deal with many variables (i.e. more variables than cases). Furthermore, some techniques are particularly adept at detecting nonlinear effects as well as differences in effects across groups [47]. The disadvantage of (some of) these models is that they may produce biased parameter estimates (yet improve out-of-sample prediction) because these models resolve the bias-variance trade-off differently [48]. Another disadvantage is that for some techniques, it is difficult to assess which variables are most important and why (although this argument is rather overblown [49]).

Here we focus on LASSO (least absolute shrinkage and selection operator) regression [48,50], which is a data-driven method that improves out-of-sample prediction, and yet provides models that are interpretable (at a small potential cost of bias in the regression estimates). Like linear regression, LASSO regression minimizes the sum of squares of the distance between the model predictions and actual outcomes but it adds a penalty term that limits the magnitude of the coefficients. LASSO regression ‘shrinks’ less important variable coefficients (in terms of predictive ability) to zero and only includes the most important variables. This form of regression, as opposed to traditional linear regression, can handle the inclusion of many variables (even more variables than data points), as well as the inclusion of many correlated variables (multicollinearity). In sum, LASSO regression helps in selecting the most important variables and leads to a sparse and interpretable model, while at the same time it prevents overfitting and improves predictive ability.

LASSO regression is also valuable for understanding personal network effects on fertility outcomes. Networks encompass diverse compositional and structural characteristics [32,51], often interrelated [32,51]. LASSO regression effectively addresses this by simultaneously modelling these variables, and many other correlated variables, while identifying the most influential, independent predictors. This approach can contribute to ongoing fertility debates. For example, theories on the demographic transition propose that the reduction in densely connected, kin-rich networks is one explanation for the decline in fertility. Often, this literature is less clear about the distinction between the density of the networks and the number of kin in these networks [52], that may be closely correlated [51]. Simultaneously modelling multiple characteristics also prevents researcher degrees of freedom in presenting a particular network variable associated with a variable of interest [53]. This favourite explanatory variable may only be ‘significant’ in a few models with particular sets of control variables and may not substantially add to predictive accuracy, risking a less empirically robust literature [54].

### 1.3. This study

In this study, we use data from a representative sample of over 700 Dutch women who each reported on 25 people in their network. Many characteristics are assessed about these people, including whether they had children, wanted to have children, or wanted to be childfree. The size of the personal networks and the large number of alter characteristics allow us to reliably calculate many compositional and structural features of the network. We use LASSO regression to examine how well we can predict five different outcomes relating to fertility preferences, and which variables are most important in explaining these different outcomes.

This study contributes to the existing literature in several ways. First, it uses a unique dataset on large personal networks from a study on a representative sample of women that was designed to examine social influences on fertility [37]. Second, we focus on out-of-sample predictive ability which is an easy-to-interpret and comparable measure of effect size. Third, we use machine learning techniques (i.e. LASSO regression and cross-validation) to robustly derive which variables are most important in explaining fertility preferences, and we compare individual-level variables, compositional variables, and structural variables. Machine learning techniques in demography are still relatively rare [38,41,55], despite the tremendous potential they might have for the field [38,41,47,56,57].

## 2. Methods

### 2.1. Sample

We make use of data from the LISS (Longitudinal Internet Studies for the Social Sciences) panel administered by Centerdata (Tilburg University, The Netherlands). This is a representative sample of Dutch individuals who participate in monthly Internet surveys. The panel is based on a true probability sample of households drawn from the population register by Statistics Netherlands (CBS). Only households in which at least one household member spoke Dutch are included. Households that could not otherwise participate are provided with a computer and Internet connection. Ten core surveys are administered in the panel every year, covering a large variety of topics. The representativeness of the LISS panel is similar to those of traditional surveys based on probability sampling [58,59]. Initial selection biases were substantially corrected by refreshment samples, and further refreshment samples were planned for attrition biases [60].

### 2.2. Social networks and fertility survey

The LISS panel allows researchers to do their own survey within the panel. We added a study named the Social Networks and Fertility survey (for further details, see [37,61,62]). This research investigates social influences on how many children people have or would like to have and when. Early 2018, all women between 18 and 40 (*N* = 1332) were invited to participate. In total, 758 women completed the survey with a mean age of 29.2 (s.d. = 6.5). Respondents were similar to non-respondents on a range of measures that are collected for all respondents [61]. Ethical approval was obtained through the ethical committee of sociology at the University of Groningen. Data and codebooks can be found at [61]. We used the R [63] package FertNet [62,64] to process the data, and igraph [65,66] and tidygraph [67] for calculating network characteristics. R-code to produce the results in the current paper can be found in [68].

### 2.3. Procedure

Respondents were invited to participate in a study on ‘social networks and fertility’ and received €12.50 for completing the survey. The first block of questions was about respondents themselves, including fertility outcomes. The second part of the questionnaire involved generating 25 names. Respondents were asked to list 25 individuals 18 years or older with whom they had contact in the last year, and instructed that it was important to list exactly 25. In total, 738 respondents (97%) listed exactly 25 alters.

Twenty-five alters were chosen because it is easy for people to name that many individuals [69,70] and this size is large enough to consist of weaker ties [71]. Networks that are smaller than 25 individuals can reduce the reliability of estimates of the network's structure and composition [35,36].

Subsequently, for each of the 25 listed individuals, 16 characteristics were assessed. We used all these characteristics as the basis for all compositional network characteristics (table 1). An important question concerned the type of relationship, with the choice of partner, parent, siblings, other relative, relative of partner, acquaintance/friend of partner, from primary school, from high school, from college/university, from work, from a social activity, through a mutual acquaintance/friend, from the neighbourhood, and other. These categories were reduced to kin (including parents, siblings, other kin, and in-laws) and non-kin. A subsequent question on whether the 25 alters were considered friends or not further allowed the non-kin to be divided into friends and non-friends. The final question about the alters concerned whether the alters had contact with one another. These alter–alter ties were at the basis of all structural network characteristics.

**Table 1.** Descriptive statistics (mean ± standard deviation or %) for all predictor variables used in the LASSO regression. Further descriptive statistics for all these variables and imputed variables can be found in electronic supplementary material, table S1.

ego variables	composition variables [continued]	structural variables
age [29.13 ± 6.49]	avg closeness kin [4.06 ± 0.63]	density [0.24 ± 0.11]
highly educated [49%]	avg closeness friends [3.92 ± 0.53]	… among kin [0.68 ± 0.24]
monthly income [1227.65 ± 993.2]	avg closeness has child [3.35 ± 0.7]	… among friends [0.32 ± 0.24]
has partner [73%]	avg closeness wants child [3.82 ± 0.81]	… among with children [0.32 ± 0.26]
no. children [0.65 ± 1.04]	avg closeness childfree [3.36 ± 1.06]	… among want children [0.39 ± 0.32]
no. women [16.1 ± 3.26]	avg closeness can help [4.33 ± 0.55]	… among childfree [0.39 ± 0.41]
**composition variables**	avg closeness can talk to [4.38 ± 0.61]	… among can talk to [0.43 ± 0.31]
no. older [15.32 ± 4.46]	avg f2f contact kin [2.93 ± 0.85]	… among can help [0.49 ± 0.27]
no. highly educated [12.01 ± 6.55]	avg f2f contact friends [3.03 ± 0.85]	no. small communities [3.7 ± 3.29]
no. kin [8.81 ± 4.44]	avg f2f contact has child [2.63 ± 0.79]	no. large communities [2.94 ± 0.92]
no. friends [10.42 ± 5.3]	avg f2f contact wants child [3.05 ± 1.05]	modularity [0.39 ± 0.17]
no. has children [8.96 ± 6.57]	avg f2f contact childfree [2.74 ± 1.21]	largest component [18.46 ± 5.59]
total no. children [18.16 ± 14.33]	avg f2f contact can help [3.44 ± 0.82]	diameter [3.44 ± 1.27]
no. children under 5 [3.74 ± 3.84]	avg f2f contact can talk to [3.66 ± 0.97]	avg normalized betweenness [0.02 ± 0.02]
no. less happy after child [0.33 ± 0.98]	avg other contact kin [3.01 ± 0.8]	avg normalized closeness [0.41 ± 0.17]
no. want child [4.66 ± 4.13]	avg other contact friends [3.29 ± 0.72]	avg normalized eigenvalue [0.4 ± 0.11]
no. childfree [1.41 ± 1.84]	avg other contact has child [2.52 ± 0.82]	no. cliques [12.57 ± 5.31]
no. can help [8.84 ± 5.3]	avg other contact wants child [3.23 ± 0.99]	no. components [4.04 ± 2.97]
no. can talk to [6.94 ± 5.88]	avg other contact childfree [2.65 ± 1.19]	betweennes centralization [0.25 ± 0.2]
avg closeness [3.48 ± 0.47]	avg other contact can help [3.57 ± 0.76]	degree centralization [0.35 ± 0.17]
avg f2f contact [2.86 ± 0.6]	avg other contact can talk to [3.87 ± 0.82]	
avg other contact [2.83 ± 0.58]		

### 2.4. Outcomes

**Figure 1.**
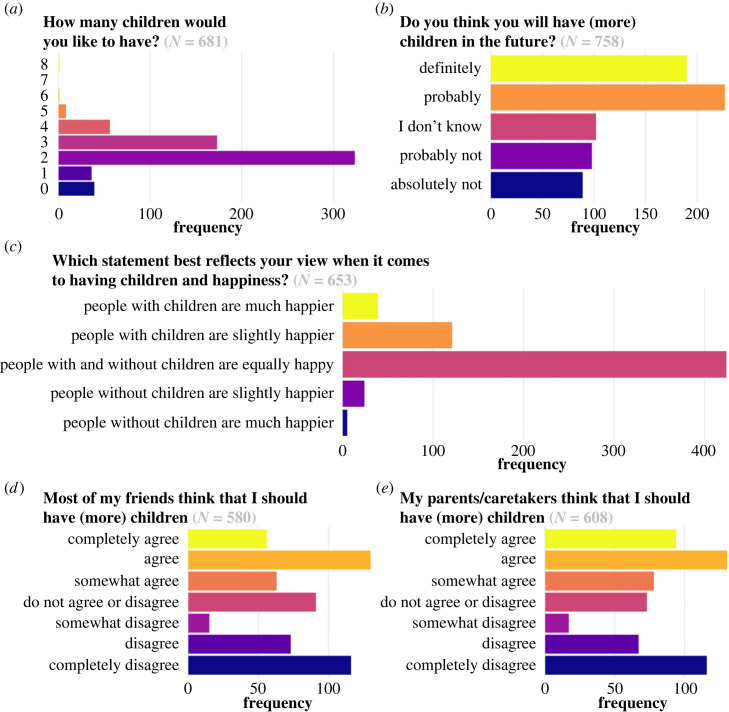
The five outcomes that models had to predict.

There were five outcomes that models predicted that we collectively refer to as fertility preferences, although it is clear that not all outcomes are captured well by this term (figure 1).

#### 2.4.1. Ideal number of children

Respondents were asked ‘How many children would you like to have? This is including the X children you already have’. They could answer anything between 0 and ‘10+’ and ‘I don't know’. We excluded respondents who said ‘I don't know’ (77 in a total sample of 758). This variable was turned into a numerical variable ranging from 0 to 8.

#### 2.4.2. Children likely in future

Respondents were asked: ‘Do you think you will have (more) children in the future?’. They could answer with: absolutely not, probably not, I don't know, probably, definitely. This variable was turned into a numerical variable ranging from 1 (absolutely not) to 5 (definitely).

#### 2.4.3. Happiness and children

Respondents were asked: Which statement best reflects your view when it comes to having children and happiness? They could select the following options: people without children are much happier than people with children; people without children are somewhat happier than people with children; people with and without children are equally happy; people with children are somewhat happier than people without children; people with children are much happier than people without children; I don't know. We excluded respondents who said ‘I don't know’ (105 out of 758), and turned this into a numerical variable from 1 (people without children much happier) to 5 (people with children much happier).

#### 2.4.4. Pressure to have children from friends

Respondents were asked: To what extent do you agree with the following statements: most of my friends think that I should have (more) children. Answer options were: completely agree; agree; somewhat agree; neither agree nor disagree; somewhat disagree; disagree; completely disagree; I don't know. We excluded respondents who said ‘I don't know’ (177 out of 758) or had missing values (1).

#### 2.4.5. Pressure to have children from parents/caregivers

This question was the same as the previous one, except ‘most of my friends’ was replaced by ‘my parents/caretakers'. There was one additional answer category ‘not applicable’. We excluded respondents who said ‘I don't know’ (103) or ‘not applicable’ (46) or who had missing values (1 out of 758). Both pressure variables were turned into a numeric variable going from 1 (completely disagree) to 7 (completely agree).

### 2.5. Predictor variables

#### 2.5.1. Ego variables

We included the following personal characteristics (or ego variables): age, the number of children the respondent had, whether the respondent had a partner, whether the respondent had high education (i.e. higher vocational education or higher), and net monthly income (in euros).

#### 2.5.2. Network composition variables

The first set of network composition variables was based on the tie strength variables—closeness to the alter, frequency of face-to-face contact, and frequency of other forms of contact with the alter—inspired by the idea that close ties may provide different resources from weak ties [23,71–73]. We computed the average for each tie strength measure across the 25 alters.

The second set of variables included counts of particular groups of people in the network that are important in previous literature [8,9,12,25,74]: the number of kin (which included parents, siblings, other kin, but also in-laws), the number of friends, the number of people with children, the number of people who wanted to have children, the number of people who wanted to be childfree, the number of people who could help with child care, and the number of people the respondent could talk to about having children. We also included a total count of children across all alters, considering pregnant alters as having a child and excluding the respondent's parents' children. Additionally, we calculated the total count of children under 5 years old across all alters, excluding the respondent's parents’ children.

The third set of network composition variables combines the first two sets. For each group (kin, friends, people with children, people who wanted children, people who wanted to be childfree, people who could help with child care, and people with whom having children could be discussed), we calculated the average closeness, average face-to-face contact, and average other forms of contact. Table 1 contains an overview of all variables.

#### 2.5.3. Network structure variables

Structural variables are derived from the relationships within the ego network, excluding the respondent. While many characteristics could be computed for each personal network, we have focused on structural features that have been theoretically or empirically significant in prior personal network analyses. We will provide brief explanations of these structural variables.

The first variable we considered is *density*, calculated as the number of relationships between individuals in the network divided by the total potential number of relationships. Density is one of the few structural characteristics that has been associated with fertility outcomes [11,30,31]. We also calculated density among particular groups, using the same groups defined above for the composition variables.

In addition to density, centrality measures are important for understanding how well-connected alters are in a network. *Degree* centrality shows how many ties each alter has, while *betweenness* centrality measures the importance of a particular alter if other alters need to communicate with each other (i.e. whether the paths between two alters in the network often go through one particular alter). *Closeness* centrality assesses the efficiency of information flow for a specific alter by calculating the shortest path to all other alters. Finally, *eigenvector* centrality measures an alter's connection to influential nodes. We calculated these indicators for each alter, and then took their normalized averages to characterize the overall network. Average normalized closeness centrality cannot be calculated for networks that include people who are not connected to anyone else, which is why we calculated the average harmonic centrality [75]. These network features could impact the transmission of novel information, promotion of shared norms and social pressure to conform to these norms. The average normalized betweenness centrality is identical to density, which is already included in the model. The inclusion of the above variables was based on [76].

We also included a group of structural characteristics that are related to specific patterns of clustering among people in the network, as a particular density and average centralities may arise from networks with very different structures [77]. Vacca [51] suggests that the structure of personal networks can be best described by three variables: the number of subgroups of size 1 or 2 (people or duos who are not connected to any other (groups of) people in the network), the number of (cohesive) subgroups of 3 and higher (interconnected groups of individuals with limited connections to other groups), and the modularity between the subgroups.

Modularity refers to how densely connected the subgroups are relative to the connections between groups. High modularity indicates that subgroups are densely connected among themselves but sparsely connected to each other. This suggests the presence of distinct groups of people in the network that may have varying ideas and values regarding fertility. To determine the subgroups in personal networks, the Girvan–Newman community-detection algorithm is used [78].

Bidart *et al.* [79] further add the proportion of alters in the network that are part of the largest component subgroup (a subgroup in which each alter is connected directly or indirectly (through other alters) to every other alter in that subgroup) and diameter—which refers to the maximum number of ‘steps’ alters need to be in contact with other alters in the network.

Additionally, Maya-Jariego [76] uses the following structural characteristics: the number of cliques (the number of groups of 3 or more in which all group members have ties among each other) and the number of components (the number of groups that are completely disconnected from the rest). The distinction between cohesive ties (ties within a clique or another tightly knit community) and the periphery ties (ties between alters that are disconnected from other groups of alters) is often found important as these different structural connections might serve different roles: cohesive ties provide support and resources, and periphery ties provide new information [71,80].

Finally, we include two measures of centralization that compare the centrality of the network's most central node to the centrality of all other nodes (based on [76,79,81]). These were *degree centralization* and *betweenness centralization* [82] which are calculated by the sum of the differences in centrality (either degree or betweenness) between the most central node and all other nodes and dividing this sum by the theoretically largest sum of differences in a graph of that size. A high centralization implies that there are some alters that play a central role in the network of the respondents because they are connected to many other alters.

There are large correlations among our network variables, because some conceptualizations will measure similar aspects of the network [32,76], for example, the number of subgroups (communities), components, and cliques. Luckily, LASSO regression can handle (many) correlated variables well (see electronic supplementary material, figure S1 and table S2, for correlations between all variables).

### 2.6. Analytical strategy

#### 2.6.1. LASSO regression

LASSO regression [50] is a form of penalized regression. In contrast to ordinary least squared regression (OLS), which minimizes the sum of squared errors between observed (*y*) and predicted (*ŷ*) outcomes, penalized regression introduces a penalty term to the traditional OLS approach. In LASSO regression, the penalty is on the sum of the (absolute) magnitude of the estimates in the model (see equation (2.1); *p* refers to the number of variables). This effectively means that there is a penalty to the inclusion of more variables and the magnitude of the effects of the variables is constrained (shrinkage). An important parameter that needs to be estimated in LASSO regression is *λ*, the penalty term. When *λ* is 0, there is no penalty on variable inclusion, and no shrinkage occurs, making LASSO regression equivalent to linear regression. When *λ* is infinite, all estimates are shrunk to zero, rendering LASSO regression equivalent to an intercept-only linear regression. Finding the optimal *λ* is key, and is achieved through cross-validation (see below). Cross-validation calculates out-of-sample predictive accuracy for a range of *λ* values. Rather than choosing the *λ* that produces the highest out-of-sample predictive accuracy, it is recommended to select the highest *λ* (more shrinkage) within one standard error of the optimal *λ*. This approach results in sparser models without sacrificing predictive accuracy [48, p. 214].

2.1 $$\mathrm{Minimize}\;\overset{n}{\underset{i=1}{\sum }}{\left(y_{i}-{\hat{y}}_{i}\right)}^{2}+\lambda \overset{p}{\underset{\;j=1}{\sum }}|\beta_{j}|.$$

Continuous variables are standardized by subtracting the mean and dividing by the standard deviation. This standardization is necessary for LASSO regression because without it, an estimate's magnitude included in the penalty term depends on the scale of the variable. After standardizing, the LASSO regression estimates can be interpreted in the same way as standardized linear regression coefficients.

#### 2.6.2. Cross-validation

Out-of-sample predictive ability is calculated through cross-validation. In cross-validation, a dataset is split into ‘training’ and ‘test’ data, such that the training data are used to build the model, and the test data are used to assess the out-of-sample predictive accuracy. Here, this performance is assessed by the out-of-sample *R*^2^. This is calculated by the formula

2.2 $$1-\frac{\sum_{i=1}^{n}{\left(y_{i}-{\hat{y}}_{i}\right)}^{2}}{\sum_{i=1}^{n}{\left(y_{i}-{\bar{y}}_{\mathrm{train}}\right)}^{2}}\;.$$

The upper boundary is 1 (all outcomes in the test data are correctly predicted). Note that the lower boundary is not 0. A negative *R*^2^ happens when out-of-sample predictions from a model including some variables are worse than those from a null model (the average of the outcome in the training data). In the electronic supplementary material, we also provide mean squared errors (MSE) for all models.

A downside of having only one split (e.g. 50% training data, and 50% test data) is that it reduces the size of the dataset for training the model and hence the quality of the model. This is particularly pertinent when sample sizes are low [49]. In this case, *k*-fold cross-validation can help. Imagine a *k* of 10: the dataset is split into ten folds. First, the first 9 folds are used as training data, and the tenth fold is used as test data. Subsequently, the first eight folds and the tenth fold are used as training data, and the 9^th^ is used as test data. This process is continued until all 10 folds have been used as test data. The overall performance is measured by the average *R*^2^ across all folds. *k*-fold cross-validation is useful because it capitalizes on the entire dataset, so in the end all cases are used as training data. We use 10 folds in this study. Cross-validation serves two purposes here. First, to determine the penalty term (*λ*) for the LASSO regression for all fertility preferences. Second, to calculate the performance of our model after *λ* has been set.

The LASSO regression model including all variables is compared to (1) the linear regression model including all variables, and to LASSO models including (2) only ego variables, (3) only network composition variables, and (4) only network structure variables. To facilitate comparison across these models, we use the same penalty term in the LASSO regression for a particular outcome (but the penalty terms differ across fertility preferences). Through cross-validation, all models are applied to predict each fold.

#### 2.6.3. Imputation on network variables

Several network variables had numerous missing values. For instance, many respondents reported having no childfree individuals in their network. For these respondents, we could not calculate average group-specific tie strength (e.g. average closeness to childfree individuals) or group-specific density (calculating density requires at least two individuals). As some people did not have kin in their networks, others no friends, yet others no childfree people, the sample size would significantly decrease if we were to exclude all respondents with missing values (only 109 would remain). This approach would also introduce bias to our sample, as it would include only respondents who listed more than two childfree individuals, more than two kin, more than two friends, etc., in their network. Instead, when values were missing because particular groups of people were missing in the network, we imputed a score of 1 for the tie strength variables (meaning the lowest possible closeness or lowest possible frequency of contact), and a score of 0 for density (no ties exist).

#### 2.6.4. Sensitivity analyses

We perform several robustness checks. First, we reran all models but excluded the network variables with missing values (i.e. excluding variables that were calculated for particular groups of alters). Second, we reran our models but only included variables with fewer than 50 missing values. To get further insight into our results, we performed two additional analyses. First, we compare the results of the model with all variables to a model in which we include all network characteristics but exclude ego characteristics. Second, we repeated the analyses for twelve different subgroups of women: three different age groups, three income groups, two educational levels, having a partner or not, and having children or not (see electronic supplementary material). All results from the sensitivity analyses are presented in the electronic supplementary material, but general patterns will be discussed in the results section.

#### 2.6.5. Sample selection

For this study, we only selected respondents that listed 25 alters as instructed. We excluded respondents who gave problematic responses to alter relationship questions, who did the survey on their phone (against explicit instructions), who had more than 10 missing values on alter attributes, and one respondent who reported no alter–alter ties (see [9]). This led to a sample of 706 women. For each outcome measure, we also excluded respondents who gave answers that could not be turned into numerical values (e.g. ‘I don't know’).

## 3. Results

### 3.1. Predictive ability

**Figure 2.**
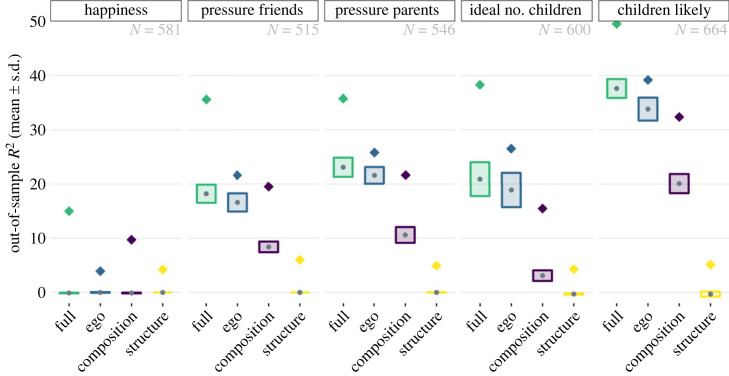
Predictive ability (*R*^2^) for different models (*x*-axis and colours) on different outcomes (panels). Full: model including all variables; ego: model including only ego characteristics; composition: only network composition variables; structure: only network structure variables. The dot is the average out-of-sample *R*^2^ from a LASSO regression across ten folds; the maximum and minimum heights of the bars represent one standard error above and below the average. The standard error is based on the standard deviation in *R*^2^ across the ten folds divided by the square root of ten. The diamonds represent in-sample *R*^2^ based on a linear regression.

Using LASSO regression including 62 variables, we were able to predict fertility preferences reasonably well, with an out-of-sample *R*^2^ between 18% and 39% (figure 2). The perception of how happiness is associated with having children was an exception with 0%, implying that even the best-performing model could not make any good predictions above and beyond a null model.

For all outcome measures, ego characteristics were the strongest predictors. Variables on network composition were also important, whereas variables on network structure hardly had any predictive ability. For example, the LASSO regression including all variables trying to predict whether people thought they were likely to have children had an *R*^2^ of 39% (figure 2). Including only ego characteristics reduced this to 35%, whereas including only network composition variables reduced this to 21%. A model with only structural network characteristics had a predictive ability of 0%, and was actually worse than predicting the mean of the training data.

If we run linear regressions including all variables for each outcome variable on the entire sample, we find in-sample *R*^2^ values between 15% and 50% (around 12–17 percentage points higher than their out-of-sample counterparts), clear examples of overfitting (figure 2). This comparison can highlight potential overoptimism in linear regression versus LASSO regression. For instance, in predicting the perceived happiness of having children based on five ego variables, out-of-sample *R*^2^ is 0, and the linear regression in-sample *R*^2^ is 4%. This linear regression identifies two significant variables (a *p*-value of 0.002 for the effect of income and a *p*-value of 0.015 for the effect of the number of children; see electronic supplementary material, table S5). In the classical statistical inference framework, this may have led to the conclusion that there are two variables important in explaining people's perceptions on how having children relates to happiness. This seems overoptimistic at best and simply wrong at worst.

### 3.2. Which variables are important?

**Figure 3.**
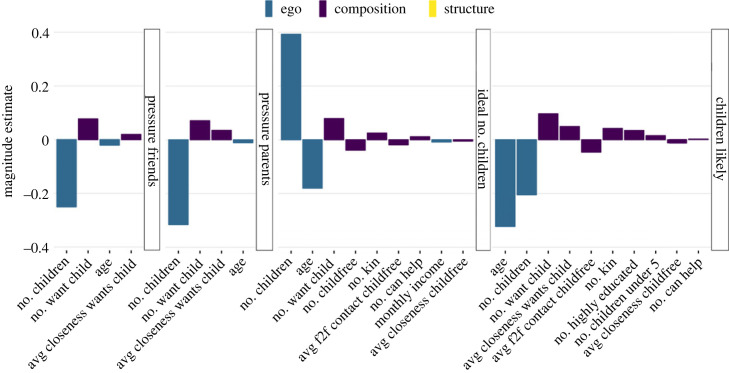
The magnitude of the LASSO regression coefficients that were not shrunk to zero in the model including all variables. For none of the outcomes, structural characteristics were kept in the model. No characteristics were kept in the model in predicting happiness in relation to having children.

With respect to whether respondents thought they would have *children in the future*, 10 variables were kept in the model (and the remaining 48 were shrunk to zero). The strongest effects were the negative effects of two ego characteristics: number of children and age (figure 3). Other negative effects, but much smaller in magnitude, were the average face-to-face contact and the average closeness towards childfree individuals in the network. Thus, stronger ties to childfree people decreased the idea of having children in the future. The strongest positive effects were found for the number of people who want children, the average closeness towards these people, and the number of kin in the network. Weaker but still positive effects were found for the number of highly educated individuals in the network, the number of children under five that alters have, and the number of people that could help with care for children.

Concerning the *ideal number of children*, nine variables were kept in the model. Again, the number of children and age were the most important variables. Women with higher incomes also had lower ideals, but the magnitude of the effect was small (figure 3). Three network composition variables had a positive effect, namely the number of people who want children, the number of kin, and the number of people who could help with the care for children. There were also three network composition variables that decreased the ideal number of children, namely the number of childfree people in the network as well as both the frequency of face-to-face contact and the closeness to these people.

In terms of whether respondents *perceived pressure from friends and parents/caretakers* to have children, results were very similar: only four variables were kept in the model. The most important variable was the number of children, which decreased perceived pressure. Older women also felt less pressure. The number of people in the network who wanted children and the closeness to these people increased perceived pressure.

In terms of whether respondents thought that *people with or people without children were happier*, no variables were retained in the final model.

### 3.3. Sensitivity analyses

When we investigated different sample selections (e.g. using only 34 variables without any missing values or including 52 variables each with fewer than 50 missing values), we observed consistent results in terms of predictive ability. The complete model yielded approximately 40% predictability for the perceived likelihood of having a child, 0% for happiness and having children, and around 20% for the other three outcomes (see electronic supplementary material, tables S3 and S4 and figures S2 and S4).

We also performed an analysis including all network characteristics while excluding ego characteristics. This analysis confirmed that structural characteristics are not important predictors of any outcome (see electronic supplementary material, figure S6). Some compositional variables that are kept in the model without ego variables were not included in the full model with ego characteristics. Those with the largest magnitude are the number of people in the network with children, and the total sum of children that alters have. The absence of these variables in the models with ego characteristics may be explained by associations between these network characteristics and the age and the number of children of the respondents (see also electronic supplementary material, table S2 and figure S1, for correlations between all variables).

Examining how results varied for different subgroups of women (e.g. younger respondents, highly educated respondents, childless respondents), we again find for nearly all models that ego characteristics are more important than network variables, and that structural variables matter little for all outcomes (see electronic supplementary material, figures S7 and S8). Network variables were more predictive than ego variables in only 3 out of 40 cases where predictive ability was at least 1%. Nevertheless, the predictive value of network variables varied across subgroups, and for a few subgroups, even structural variables were retained. For example, for older women in our sample (between the ages of 35 and 41), several network structure variables predicted perceived pressure from friends, including the density among people who have children and the density among people who would not like to have children. For younger women, network variables had no predictive value.

## 4. Discussion

In this study, we used a data-driven approach to assess the impact of various compositional and structural network characteristics on five outcomes related to fertility preferences. Almost all outcomes were reasonably well predicted. Roughly 40% of out-of-sample variation in the perceived likelihood of having children in the future could be predicted by variables about individuals and their networks. This was about 20% for the ideal number of children and for the perceived pressure to have a child from both friends and parents/caretakers. By contrast, individuals' ideas on whether people's lives are happier with children could not be predicted at all. Contrasting out-of-sample predictive ability with in-sample predictive ability shows that the latter is about fifteen percentage points higher than the former. This is a clear case of overfitting, and in-sample predictive ability thus gives a rather misleading picture of our ability to predict (and understand) fertility preferences [49], and may lead to overconfident or erroneous conclusions.

It is difficult to assess whether our estimates of out-of-sample predictive ability are high or not compared to other demographic outcomes, given that predictive ability is rarely assessed within demography and the social sciences more broadly [40,41]. This is unfortunate, because predictive ability is a useful, comparable measure of effect size that allows comparison across statistical techniques and across theory- versus data-driven methods. Data-driven methods can assist theory-building by being better able to detect robust, novel predictors, nonlinear effects and differences in effects across groups [47]. The data-driven estimates of predictive ability that we have established are further useful because they can be seen as prediction benchmarks: others will be able to assess whether their statistical approach or their inclusion of additional variables based on theory will improve predictive ability. These benchmarks promote cumulative progress [49] and are considered a key component in the major success of data science [83], exemplified by the ImageNet challenge [84]. This competition in image classification spurred remarkable advances in computer vision over the years, ultimately reducing the error rate from 25% to 2.5%, surpassing human performance [49].

Our data-driven approach focusing on out-of-sample predictive ability allowed us to systematically analyse five different outcomes and thus get insight into the similarities and differences in predictive variables across these different outcomes. From this analysis, it was clear that individuals' characteristics were more important than their networks in predicting fertility preferences. Among network characteristics, only variables related to composition were predictive, whereas variables on network structure were not. Particularly people in the network who would like to have children and those who would like to be childfree, and the strength of relationships to these people, were important across outcomes.

Although our results consistently showed that individuals’ characteristics are more predictive than their networks across different outcomes, different robustness checks, and different subgroups of women, our analyses also showed that the predictive value of particular network characteristics varied across subgroups (e.g. women with and without children). Future research may better tap into these nonlinear effects and interactions between variables [47]. Data-driven methods like random forests [85] are better able to detect such patterns than LASSO regression. The predictive ability of such methods can also be directly compared to those reported here.

LASSO regression results in sparse models including only the most important variables in terms of predictive ability. As such, it does not give insight into causal processes. This is a major limitation of this particular approach, although we argue that the prevailing statistical practices of focusing on the signs and significance of coefficients of a small set of variables based on a simple theoretic model chosen because of either cognitive or feasibility constraints cannot establish causality either. Sophisticated research designs using instrumental variables or quasi-experimental designs have been able to assess causal effects of social interactions on outcomes related to fertility [12,86,87]. Establishing causality in network studies is particularly difficult [88–90], because in addition to common background variables, people select individuals into their network [91], and are influenced by them. For example, people who would like to be childfree may be (more) likely to surround themselves with like-minded individuals, reinforcing their childfree sentiments. Longitudinal network data are a first step towards disentangling the effects of selection and influence.

Thus, the descriptive findings presented here cannot be understood in causal terms, although the variables that were identified as important are a good starting point for finding causes. For instance, the finding that individuals in the networks that desire children and that desire to be childfree, and the relations to these individuals are important in predicting several outcomes, suggest that these groups of individuals may be central to people's reproductive decision-making, either through strengthening relationships to similar-minded individuals or because of influence from these individuals on people's preferences. Furthermore, these characteristics of individuals in the network are likely more important for fertility than their demographic make-up (e.g. sex, age) that was not predictive of any outcome, and may deserve further inquiry in comparison to, for example, kin that are well represented in this field of research but whose impact in our analyses was limited.

With the caveat of causality in mind, we can attempt to characterize the impact of network composition characteristics on fertility preferences through the lens of four social mechanisms: social learning, social support, social pressure and social contagion. The number of people in the network who did not have children, but who wanted to have children, was important for most fertility preferences: it increased perceived pressure, and it led to increased pro-natal preferences (i.e. higher ideal number of children, higher perceived likelihood of having children in the future). The closeness people felt towards these people who wanted to have children was further important, with higher closeness leading to stronger pro-natal preferences and increased perceived pressure. The closeness and frequency of contact with people in the network who wanted to remain childfree, in contrast, decreased pro-natal preferences. These results are probably best seen as evidence for social learning, where the ideas and preferences of other people shape one's own ideas and preferences.

The finding that the perceived pressure to have children is increased by those people who want children but not decreased by those who want to remain childfree may be explained by prevailing cultural norms surrounding fertility. Having children is still considered normative, with the choice of wanting to remain childfree facing more resistance [92]. In such a setting, the pressure that childfree individuals need to exert to convince others to break prevailing norms will be higher.

We also found some evidence that social support matters for fertility preferences. For both the ideal family size and the perceived likelihood of having children, we found that the number of people in the network who can help with raising a child increased pro-natal perceptions. Similarly, the number of kin in the network increased pro-natal perceptions (see also [6,9,16,27]), which is likely due to the increased support kin can give, although these kin influence effects have also been attributed to social learning and social pressure.

The finding that the number of children under five increases women's perception of having children in the future could be interpreted as social contagion. However, social contagion is nearly impossible to disentangle from social learning (see also [93]), the difference being whether the influence happens consciously versus subconsciously [17].

No independent effects of network structure were found. This is at odds with research arguing for the important role of network density in imposing norms and spreading novel information [17,31,94]. Density is correlated to many network composition variables, and this is exacerbated in small networks. As an example, density is associated with average frequency of contact and closeness of relationships, owing to the process of triadic closure commonly observed in social networks [95,96]. This means that if a person has two close contacts, these contacts are likely to know each other. Previous studies may have erroneously concluded that density was important for fertility outcomes, given that such studies typically ignore various compositional measures and are based on small(er) networks. A further reason for this discrepancy in results is that previous studies have shown that the effects of density are statistically significant, but significant does not mean important or predictive [97]. We show that density, when controlling for network composition, is not important in terms of predictive ability. These findings call for a revision of our knowledge of the effects of density on fertility outcomes and studying the mechanisms more closely.

For all outcomes, personal characteristics had a more substantial impact on fertility preferences than network characteristics. Particularly the respondents' number of children and age had strong effects on their perceptions related to fertility. However, this does not imply that networks are unimportant. First, some of the personal characteristics may be a consequence of earlier socializing effects. For example, the number of children an individual has may itself be a consequence of network effects earlier in life, or even socializing effects during childhood. Second, personal and network characteristics might not be easily separated. For instance, age had a strong effect on all fertility preferences. The composition and structure of networks change across the lifespan. If various aspects of the network change with age, some of which we may not have captured with our network variables, our statistical models may include age in favour of other network variables even though the reason age is included is exactly because it is tied to many network characteristics. Third, network characteristics are more difficult to measure and will have more measurement error and sampling variation than personal characteristics. For example, reporting on whether two people in the network have contact with one another (which needed to be assessed for 300 relationships) generates more measurement error than the age and number of children of the respondent.

Our prediction-centred approach offers the advantage of accommodating a large set of (correlated) variables in the models without overfitting the data and identifies the most important variables based on their predictive performance. Further research using longitudinal data could delve into potential mechanisms, starting with the most important predictive variables. In this case, there could be more research into the influence of people who would like to have children as well as those who would like to be childfree, both of which have rarely been studied.

## Data Availability

Data from this study can be accessed here: https://www.dataarchive.lissdata.nl/study_units/view/1377. The data are freely available but registration is required. Materials to reproduce the findings in the current paper in addition to supplementary materials can be found in [68].
